# Job Satisfaction among Medical Doctors in Nepal: A Descriptive Crosssectional Study

**DOI:** 10.31729/jnma.8404

**Published:** 2024-05-31

**Authors:** Jeevan Gyawali, Kritika Mishra, Bhim Chauhan, Agnimshwor Dahal, Binita Lamichhane, Bijaya Shrestha, Nejina Rijal, Bishrut Sapkota, Ajit Kumar Sah, Sneha Shah, Madhusudan Subedi

**Affiliations:** 1 Dailekh District Hospital, Narayan Municipality, Dailekh, Nepal; 2 Nepal Intensive Care Research Foundation, Kathmandu, Nepal; 3 Ilam Hospital, Ilam, Nepal; 4 Himal Hospital, Kathmandu, Nepal; 5 Patan Academy of Health Sciences, Lalitpur, Nepal; 6 Kathmandu Medical College Teaching Hospital, Kathmandu, Nepal; 7 Kantipur Hospital, Kathmandu, Nepal

**Keywords:** *healthcare*, *healthcare policies*, *job satisfaction*

## Abstract

**Introduction:**

Job satisfaction is an attitudinal variable representing the extent to which people like or dislike their jobs. It is a critical factor influencing healthcare quality, patient outcomes, and overall well-being in medical professionals. This study aimed to determine Job Satisfaction among medical doctors in Nepal.

**Methods:**

A descriptive cross-sectional study was conducted among medical doctors of Nepal between 11 June 2023 and 31 August 2023 after obtaining ethical approval from the Ethical Review Board of Nepal Health Research Council. A convenience sampling method was used. The point estimate was calculated at a 95% Confidence Interval.

**Results:**

Among 380 participants, 63 (16.58%) (12.84-20.32 at 95% Confidence Interval) expressed satisfaction. The number of satisfied participants above 40 years were 10 (16%) and less than 40 years were 53 (84%). Out of 63, 46 (73.02%) were male and 17 (26.98%) were females.

**Conclusions:**

Job satisfaction among doctors practicing in Nepal was found to be lower than the studies conducted in similar settings.

## INTRODUCTION

Doctors play an important role in delivering quality healthcare services, and their satisfaction with their job is closely linked to the overall effectiveness and efficiency of the healthcare system.^1^ It is a pleasurable or positive emotional state resulting from a positive appraisal of one's job or job experience influenced by extrinsic (salary, security, safety) and intrinsic (recognition, performance measurement) factors.^2^ High job satisfaction boosts health-related quality of life, performance, retention, healthcare quality, and patient contentment, whereas low satisfaction leads to imbalance, turnover, errors, stress, and doctor emigration.^1^

Nepal needs health workers as with 0.17 doctors per 1000 the situation is clearly an overburdened workforce. Nepal Medical Council (NMC) receives four daily verification requests for foreign jobs.^1^ In the context of Nepal, a study on doctors' job satisfaction can contribute valuable insights for policy formulation and organizational intervention.

This study aimed to find job satisfaction among medical doctors in Nepal.

## METHODS

A descriptive cross-sectional study was conducted among medical doctors of Nepal from 11 June 2023 to 31 August 2023 after obtaining ethical approval from the Nepal Health Research Council (Reference number: 418-2023). Medical doctors currently practicing in Nepal were included in the study. Convenience sampling was used. The sample size of the study was calculated using the formula:


$$\begin{array}{ll} \text{n} & ={\text{Z}}^{2}\times \frac{\text{p}\times \text{q}}{{\text{e}}^{\text{2}}}\\ & =1.96^{2}\times \frac{0.436\times 0.564}{0.05^{2}}\\ & =378 \end{array}$$

Where,

n = minimum required sample size
z = 1.96 at 95 % Confidence Interval (CI)
p = prevalence taken as 43.6% for sample size calculation ^5^
q= 1-p
e= margin of error, 5%

The minimum expected sample size was 378. However, we took 380 samples for the study.

The distribution of the survey link was shared to closed groups like Viber, Messenger, and Facebook pages within the medical fraternity.^3^ A data collection form was created in the Kobo Toolbox, incorporating the variables and question types. Their responses were rated on a five-point Likert scale ranging from 1 (Very dissatisfied) to 5 (Very satisfied).^3^ The midpoint of the scale is 2.5 in the scale. A score of job satisfaction >2.5 gives direction toward job satisfaction and a score ≤2.5 gives direction toward job dissatisfaction in overall and in all domains.^3^ The overall score is calculated from the individual 49 items. A total score of more than 120 was considered to be satisfactory. A score of more than 120 was used to classify the study participants into satisfied and not-satisfied classes.^4^ Collected data were exported from the Kobo Toolbox in XLS format. Then, it was carefully cleaned.

Data were entered and analysis was performed using Statistical Package for Social Sciences (SPSS) software. The point estimate was calculated at a 95% Confidence Interval.

## RESULTS

Among 380 participants, 63 (16.58%) (12.84-20.32 at 95% Confidence Interval) expressed satisfaction. The number of satisfied participants above 40 years was 10 (16%) and less than 40 years were 53 (84%). Out of 63, 46(73.02%) were male and 17 (26.98%) were females.

The study found that doctors in Nepal expressed dissatisfaction in all seven domains, with average scores below the midpoint of 3. The least satisfaction was reported in job privileges (average 1.79), career development (average 2.25), and human resource issues (average 2.25). The responses showed low variability, with standard deviations up to 1.18.

Out of 63 doctors, 50 (79.37%) of the study population had work experience of more than five years, and 39 (61.90%) were staying at the current place of posting for more than five years.

**Table 1:** Domains and factors of job satisfaction.

Parameter	Mean	S.D.
**Domain 1: Privileges attached with job**	1.79	0.76
Pension benefits	1.66	0.95
Housing loan facility	1.60	0.9
Provident fund/gratuity provisions	1.80	1.074
Children education assistance	1.50	0.85
Maternity and paternity benefits	1.90	1.1
Residential accommodation facility	1.80	1.09
Salary and allowances	1.90	1.08
Conveyance reimbursement facility	1.71	0.9
Leave provisions	2.20	1.15
**Domain 2: Interpersonal relation and cooperation**		2.77	0.86
Appreciation of work by seniors	2.78	1.085
Encouragement I get from the well-accomplished job	2.61	1.1
Openness in the relationships among employees	2.86	1.03
Support I get from my boss for family-related problems/issues	2.66	1.36
Senior's attitude toward the juniors	2.62	1.13
The way subordinates respect my authority	2.70	1.22
Working with co-workers	3.30	1.03
Supervision by seniors	2.800	1.06
The way discipline is imposed	2.58	1.10
**Domain 3: Working environment**	2.41	0.92
Job security	2.23	1.33
Degree of independence associated with my work roles	2.47	1.14
Retirement age for health care personnel in the organization	2.41	1.23
Keeping all parameters into consideration, overall satisfaction with working	2.34	1.11
Facility of electricity	2.65	1.18
The way insecurity is created about job among staff	2.40	1.23
Number of staff deployed/available in the health facility	2.32	1.25
Working environment	2.50	1.13
**Domain 4: Patient relationship**	2.60	0.83
Demand from the patients	2.30	1.00
Behavior of the patients toward staff	2.70	1.03
Facilities like supply of essential items/logistics required to run the health facility	2.44	1.06
Quality of care in the health facility	2.60	1.06
Implementation of health programs in the health facility	2.50	1.80
**Domain 5: Organization facilities**	2.70	0.92
Working space	2.63	1.167
Drinking water facility	2.91	1.22
Tea and coffee facility	2.34	1.22
Cooling facility in the summer	2.56	1.24
Physical working conditions of health facility	2.50	1.18
Location of health facility	3.10	1.13
Heating facility in Winter	2.53	1.20
**Domain 6: Career development**	2.25	0.94
Chance of learning new skills in the present job	2.61	1.15
Provision of training	2.31	1.12
Chance of getting official training for skill development outside the city/country	2.08	1.12
Career growth and promotions	2.00	1.0
**Domain 7: Human resource issues**	2.25	0.84
Transparency in recruitment/selection of the staff	2.05	1.01
Time taken in the process of recruitment/selection for the staff	2.10	1.0
Time spent reaching the health facility	2.65	1.12
Instructions about the job	2.58	1.06
Workload at working place	2.15	1.14
Working hours	2.14	1.18
Family and work balance	2.04	1.1

**Table 2:** Demographic and Professional Characteristics of Satisfied Doctors.

Characteristics	n (%)
**Age (years)**	
40	53 (84.13)
>40	10 (15.87)
**Gender**	
Female	17 (26.98)
Male	46 (73.02)
**Length of service (years)**	
<5	50 (79.37)
>5	13 (20.63)
**Stay in present setting (years)**	
<5	39 (61.90)
>5	24 (38.10)
**Discipline**	
Consultant	24 (38.10)
Medical Officer	39 (61.90)
**Stays with family**	
Yes	38 (60.32)
Having any post-graduate degree/diploma	
Yes	27 (42.86)

## DISCUSSION

Among 380 participants, 63 (16.58%) expressed satisfaction. This result is of concern due to the direct impact of doctors' job satisfaction on patient safety and healthcare quality.^5^ This level of dissatisfaction is considerably higher than the findings of a study carried out at Armed Forces Medical College, Pune, where only 40% of participants were dissatisfied.^6^ A systematic review showed that 59% of the physicians in European hospitals are satisfied.^7^ Research done in 1998 in Norway showed 50% of their doctors were satisfied with the job.^8^ In contrast, studies from Karachi and Sri Lanka reported 32% an 43.6% satisfaction rates, respectively.^5,9^ The enhanced advantages and perks tied to jobs, particularly salaries, could be attributed to this result in developed countries.

Our research revealed the lowest mean score among doctors with job-related benefits, with various factors contributing to this dissatisfaction with aspects such as children's education assistance, housing loan provision, pension benefits, reimbursement for conveyance, and salary allowance. Similarly, a study done at the teaching hospital of Bahawalpur showed that service structure and low income were the main factors contributing to job dissatisfaction.^10^ However, our research shows that characteristics such as working with coworkers, openness in relationships among employees, appreciation of work by seniors, and the way subordinates respect my authority scored higher than others in terms of interpersonal relationships and cooperation. Among all doctors, they expressed greater satisfaction working with their co-workers and the location of the health facility.

Our study showed that a lack of noteworthy differences in job satisfaction levels was evident across various factors, including age, gender, length of service, stay in the present setting, discipline, stay with family, and having any postgraduate degree/diploma. However, another study showed a consistent increase in job satisfaction levels with an increase in age, significantly correlated with overall satisfaction.^1^ A Norwegian study also correlated positively with increasing age.^8^ A similar study in rural West China showed better job satisfaction among female doctors.^11^ However, our study showed no difference in job satisfaction levels in male and female doctors. The yearly migration trend of our proficient physicians and medical graduates is increasing, which has caused the prevailing shortage of physicians in remote healthcare facilities. Ravi Shankar has mentioned various push and pull factors in such migration.^12^

There is a consensus among the doctors surveyed regarding their overall dissatisfaction in most aspects of the job, and there is widespread dissatisfaction observed in the data that does not correlate with physician characteristics such as age and gender. Even though there isn't a substantial notable contrast in satisfaction levels based on the length of service. This outcome contradicts the observations from studies conducted in Thailand, where individuals with longer tenures expressed job satisfaction and displayed a reduced likelihood of job turnover.^9^ Health organizations rely on practical cooperation between team members in a complex working environment. This collaboration helps in elevating the quality of healthcare. The positive attitude and encouragement from the team stand as a pivotal factor influencing job satisfaction. Our finding aligns with the outcomes of multi-center studies from other countries. These studies underscore the paramount significance of positive inter-professional relationships as a critical driver of overall job satisfaction.^13-15^

Compared to other aspects of physicians' job satisfaction, emotional exhaustion, and lower personal satisfaction also influenced predictors in both developed and developing countries.^6^ Like the outcomes of our current study, a study conducted at a tertiary care hospital in eastern India similarly discovered that doctors with postgraduate qualifications were less content compared to their colleagues with lower qualifications.^1^ The working environment profoundly impacts job satisfaction. Our research showed that all doctors were unsatisfied with the job security, retirement age, facilities like electricity, and human resources available at the workplace and as a whole working environment.

There were a few limitations in our study. Due to their demanding work schedules, obtaining a timely physician response was challenging. The participants influenced the self-responded questionnaires by recall bias. Owing to the study's cross-sectional nature, a longitudinal perspective was unattainable.

## CONCLUSIONS

Job satisfaction among doctors practising in Nepal was found to be lower than in the study conducted in similar settings. These findings can guide policymakers in devising strategies to enhance job satisfaction. The results underscore the need for frequent and regular job satisfaction surveys. The data collected from such surveys will provide insight into the workers' expectations and the employees' views regarding the various shortcomings of different dimensions at their workplaces. Policymakers can look at these results and incorporate them into health human resources policies for health that provide better compensation packages and job descriptions to employees and help improve the satisfaction of healthcare workers in the future. We advised to take an all-inclusive approach to strengthen the policies addressing employee satisfaction, which can bring a noticeable improvement in the quality and performance of the organization.
